# Outcomes of rapid response team implementation in tertiary private hospitals: a prospective cohort study

**DOI:** 10.1186/s12245-019-0248-5

**Published:** 2019-10-30

**Authors:** Awad Al-Omari, Abbas Al Mutair, Fadi Aljamaan

**Affiliations:** 1 Research Center, Dr. Sulaiman Al Habib Medical Group, King Fahad Road – Olaya, P. O. Box 301578, Riyadh, 11643 Kingdom of Saudi Arabia; 2 Alfaisal University, King Fahad Road – Olaya, P. O. Box 301578, Riyadh, 11643 Kingdom of Saudi Arabia; 3 School of Nursing, Wollongong University, Wollongong, Australia; 4 Health Sciences College, University of Sharjah, Sharjah, United Arab Emirates; 5 King Saud University, P. O. Box 301578, 11643 King Fahad Road - Olaya, Riyadh, Kingdom of Saudi Arabia

**Keywords:** Rapid response team, Cardiopulmonary arrest, Resuscitation, Mortality rate

## Abstract

**Background:**

Cardiopulmonary arrest may result in high mortality rate in hospitals where the rapid response team is not implemented. A rapid response system can recognize patients at high risk of cardiopulmonary arrest and provide the needed medical management to prevent further deterioration. The rapid response system has shown a dramatic reduction in mortality rate and cardiopulmonary arrest.

**Objective:**

To evaluate the effectiveness of the rapid response team (RRT) implementation in reducing the mortality rate, number of cardiopulmonary arrests, and number of ICU admission.

**Design:**

A pre- and post-rapid response team system implementation.

**Setting:**

Four tertiary private hospitals in Saudi Arabia.

**Patients:**

A total of 154,869 patients in the 3-year before rapid response system period (January 2010 to December 2012) and a total of 466,161 during the 2.5-year post-RRT implementation period (January 2014 to June 2016).

**Results:**

Results indicated that ward nurses activated RRT more often than physicians (1104 activations [69%] vs. 499 activations [31%]), with cardiovascular and respiratory abnormalities being the most common triggers. Serious concern about the patient condition by the ward staff was the trigger for 181 (11.29%) activations. The RRT provided a variety of diagnostic and therapeutic interventions. Most patients cared for by RRT were admitted to ICU 1103 (68.81%), and the rest 500 (31.19%) were managed in the ward. After the implementation of the RRT project, the hospital mortality rate dropped from 7.8 to 2.8 per 1000 hospital admission. Hospital cardiopulmonary arrest rate has dropped from 10.53 per 1000 hospital admissions to 2.58. Rapid response team implementation also facilitated end-of-life care discussions.

**Conclusion:**

Implementation of the RRT project has shown a dramatic reduction in the total ICU admissions, average ICU occupancy rate, total hospital mortality, and total ICU mortality. These findings reinforce the evidence that RRT implementation is effective in reducing hospital mortality and cardiopulmonary arrest rates in addition to other outcomes related to healthcare quality.

## Strengths and limitations of the study

- The study was conducted in four tertiary private hospitals with a relatively good sample size.
- The study evaluation of only adult pre- and post-ICU excluding pediatric ICU and CCU.
- The study lacks robustness to assumption as the study was conducted in private hospitals in Saudi Arabia where public hospitals may further add to the study findings.

## Background

Rapid response team (RRT) system is implemented to identify the deteriorating hospitalized patients who show clinical warning signs [1–3]. The rationale of the RRT program is to improve the safety of those hospitalized patients whose condition is deteriorating quickly. The identification of those patients is based on early notification, rapid intervention, and ongoing evaluation [4]. Patients at high risk are to be identified by the trained rapid response team that provides intervention based on each case individually and then on an ongoing evaluation of the system’s performance [4]. Regardless of the different terms being used, critical care outreach, medical emergency teams, medical response teams, and rapid response teams are the same in terms of the way of activation and the response of the team [4].

Although the rapid response system concept has been largely adopted and endorsed, evidence supporting its effectiveness comes mainly from non-randomized short-term studies where pre- and post-implementation of RRT are compared [1, 5–8]. In a study conducted at Saint Luke’s Hospital between 2004 and 2007, a total of 376 rapid response team activations were evaluated in the post-implementation period [9]. The results revealed no statistically significant differences in the primary endpoint of hospital-wide code rates and hospital-wide mortality rate. The pre-intervention and post-intervention design which was mainly adopted by most of the studies may have not adequately adjusted for the disease severity [1, 5–9].

In a recent systematic review and meta-analysis study conducted by Mahraj, Raffaele, and Wendon, they reviewed 29 studies including randomized controlled trials and pre-post rapid response team system implementation [10]. The results of the review showed a reduction in the hospital mortality rate in both adult and pediatric population. Additionally, RRT system implementation was associated with a reduction in cardiopulmonary arrests in adult and pediatric patients [10].

Furthermore, implementation of a rapid response team may skill the hospital ward staff. A hypothesis was approved by nurses surveyed in two studies which were conducted in Canada and Australia [11, 12]. Several national and international health professional organizations have recommended adopting a rapid response team concept [13–15]. These organizations included the Institute for Healthcare Improvement [13], the Department of Health in the UK [14], and the Australian Commission on Safety and Quality in Healthcare [15].

### Study design

This is a prospective cohort pre- and post-rapid response team implementation study. Data was retrieved from the pre-RRT 36-month period (January 2010 to December 2012) and was compared to post-RRT 30-month period (January 2014 to June 2016). The study was conducted in four private tertiary hospitals in total which provide general and tertiary clinical services. The 118-bed medical-surgical ICU admits approximately 1000 patients yearly and is managed as a closed unit by board-certified critical care consultants on a 24/7 in-house basis along with specialist and residents.

### Implementation of RRT

In the pre-RRT period, the response to unstable patients in hospital wards followed a traditional hierarchy starting from the bedside nurse escalating in a stepwise approach to the “first-on-call” physician, the senior resident, and then the attending physician with several cycles of evaluation and management. The ICU team involvement was typically sought later and followed a similar hierarchy.

The RRT intervention was implemented as a plan-do-study-act project with the aim of reducing unanticipated non-ICU cardiopulmonary arrests and reducing ICU morbidity and mortality. Preparation for RRT implementation started in August 2013.

RRT was structured as a system of four components: (1) The system consisted of afferent component that included the process of event detection and response triggering. (2) The efferent component was led by a trained intensivist (an ICU resident or specialist under on-call coverage of board-certified ICU consultant), an ICU nurse, and a respiratory therapist. (3) The quality improvement component involved the development of a database to monitor the RRT activations and their outcomes. (4) The administrative component consisting of the RRT committee oversaw the function of the intervention and reported to hospital administration.

The composition of the rapid response team includes primary nurse, unit charge nurse, ICU specialist, ICU nurse, respiratory therapist, ICU on-call consultant, and an on-duty nursing supervisor. RRT is led by an intensivist, and patients will be followed up for 48 h post-ICU admission. The calling criteria are based on the presence of any abnormality and derangements in vital signs. The roles of the rapid response team involve the administration of oxygen, intravenous fluids, bronchodilators, and respiratory therapy. RRT performs CPR if needed, decides whether to manage the patient in the ward or transfer them to the ICU, and facilitate the discussion about end-of-life care.

### Data collection and outcome measures

Data was obtained from the hospital information system, from the records of the cardiopulmonary resuscitation committee, and from the prospectively collected ICU and RRT databases. Outcomes to be assessed were included: we compared the pre- and post-RRT period total hospital admission, total ICU admission, average ICU occupancy rate, total hospital mortality, and total ICU mortality. We also analyzed the RRT data such as demography, RRT activation personnel (doctor or nurse), RRT triggers, RRT intervention, and mortality (patient died in the ICU and in the ward).

### Data analysis

All statistical analyses were performed using the SAS software (version 9.1; SAS Institute, Cary, NC). The Student *t* test and chi-square test were used to compare the differences between the groups. Relative risk (RR) and 95% confidence intervals (CIs) were reported for categorical outcomes. The *p* value of ≤ 0.05 was considered to be statistically significant.

## Results

The primary outcomes were hospital mortality and in-patient (non-ICU) cardiopulmonary arrest activation rate. There were a total of 154,869 hospital admissions and a total of 466,161 post-hospital admissions in the pre-RRT and post-RRT implementation, respectively. After RRT implementation, there were 1603 RRT activations which lead to a decrease in ICU admission from 44.65 to 20.70 per 1000 hospital admissions (RR 0.53; 95% CI 0.42–0.62; *p* < 0.0001).

### RRT activations and interventions

As shown in Table 1, ward nurses activated RRT more often than physicians (1104 activations [69%] vs. 449 activations [31%]), with cardiovascular and respiratory abnormalities being the most common triggers. Serious concern about a patient condition by the ward staff was the trigger for 181 (11.29%) activations. The RRT provided a variety of diagnostic and therapeutic interventions (Table 1). Most patients cared for by RRT admitted to the ICU (68.81%) and the rest managed in the ward.

**Table 1:** RRT population characteristics; RRT activations from different hospitals (*n* = 1603)

Staff activating, *n* (%)	
Nurse	1104 (69%)
Physician	449 (31%)
Gender, *n* (%)	
Male	858 (53.52%)
Female	745 (46.54%)
Activation triggers, *n* (%)	
Cardiovascular	627 (39.11%)
Respiratory	727 (45.35%)
Neurological	272 (16.97%)
Renal	21 (1.31%)
Other causes (serious concern)	181 (11.29%)
RRT intervention, *n* (%)	
Vasopressors administration	87 (5.43%)
Blood transfusion	163 (10.17%)
Increase nursing acuity	103 (6.43%)
Fluid bolus administration	682 (42.55%)
O_2_ requirement	979 (61.07%)
Disposition of RRT activations, *n* (%)	
RRT population stayed in the ward	500 (31.19%)
RRT population admitted to the ICU	1103 (68.81%)
RRT population length of stay (days)	
Average ICU length of stay	8.7
Average hospital length of stay	28.8
RRT population outcome, *n* (%)	
Discharged alive from hospital	1374 (85.71%)
Code blue upon activation	17 (1%)
Overall mortality	229 (14.29%)
RRT patients died in the ICU	216 (13.47%)
RRT patient died upon activation	13 (0.08%)
RRT patients labeled as DNR	48 (3%)

### RRT impact on the outcomes of patients on hospital wards and ICU

Non-ICU cardiopulmonary arrests decreased from 10.53 to 2. 58 per 1000 hospital admissions (RR 0.72; 95% CI 0.69–0.75; *p* < 0.0001) (Tables 2 and 3). Total hospital mortality decreased from 7.89 to 2.8 per 1000 hospital admissions (RR 0.62; 95% CI 0.59–0.65; *p* < 0.0001). ICU cardiopulmonary arrest decreased from 115.7 to 48.8 per 1000 hospital admissions (RR 0.8; 95% CI 0.70–0.88; *p* < 0.0001). The patients intubated in the ICU increased from 119 to 179 per 1000 ICU admissions (*p* < 0.0001).

**Table 2:** Characteristics of RRT population admitted to the ICU (*n* = 1103)

Major causes for ICU admission, *n* (%)	
Respiratory	513 (46.5%)
Cardiovascular	420 (38%)
Neurological	106 (9.6%)
Other causes	63 (5.7%)
Airway management	3 (0.2%)
Intubated	305 (27.65%)
Tracheostomized	128 (11.6%)
RRT patient ICU outcomes, *n* (%)	
Readmitted to the ICU	103 (9.34%)
Readmitted after 48 h of discharge	25 (2.27%)
Code blue activation	199 (18%)
Mortality in the ICU	216 (19.58%)
Discharged alive from the ICU	884 (80.14%)

**Table 3:** Outcome measures comparison in pre-RRT and post-RRT period

Parameter	Pre-RRT	Post-RRT	Absolute risk reduction CI	Relative risk reduction % CI	*p* value
ICU cardiopulmonary arrest activation number	570 (0.5%)	444 (0.1%)	0.4	80% (70.6–87.1)	*p* < 0.0001
Relative to 1000 ICU admissions	115.71	48.89	66.81	57.7	
Relative to 1000 hospital admissions	0.14	0.10	0.04	28.6	
Patients intubated in the ICU number	589 (0.5%)	1630 (0.4%)			*p* < 0.0001
Relative to 1000 ICU admissions	119.57	179.50	− 59.93	− 50	

## Discussion

This study showed a reduction in mortality rate, cardiopulmonary arrests, and ICU admission post-implementation of a rapid response team system. Although RRT is a multidisciplinary team, nurses in the current study activated the system more often than physicians. The vast majority of the patients (68.81%) in the present study got transferred to the ICU, whereas only the rest of the patients were admitted in the ward. As per the discussion, following the rapid response team implementation of the system has shown facilitation of end-of-life care discussion.

The impact of the rapid response system on ICU workflow is extremely significant. Many of the previously reviewed studies were not able to show the impact of the rapid response system activation on the number of ICU admissions [16–18]. The findings of the recent study revealed that the implementation of RRT system has been associated with a significant reduction on the number of ICU admissions from 44.65 to 20.70 per 1000 hospital admissions (RR 0.53; 95% CI 0.42–0.62; *p* < 0.0001). Nevertheless, another study found also that the number of ICU admissions was decreased after the implementation of the RRT system [19].

The present study extends the findings of other studies which investigated the reduction in the mortality rate following the implementation of the RRT system. Similarly, other studies have also shown a reduction in the mortality rate ([4, 12, 17–20]). This decline might be as a result that rapid response team transferred the patient to the ICU which was endorsed by the study findings as 1103 (68.81%) of the total patients were transferred to the ICU. Many studies have failed to show a reduction in the hospital mortality rate post-RRT system implementation [1, 5–9], and as did a recent systematic review and meta-analysis study which evaluated the mortality rate of 2,160,213 patients [10]. The overall reduction in hospital mortality rate was found in both adult and pediatric patients.

Previous studies showed that the composition of the rapid response team is uncertain [21–23]. Those three studies did not show a difference in the rapid response system led by intensivists or other team members. Moreover, most of the activations did not even require the presence of a physician [10]. The findings in the current study demonstrated that nurses activated the system more often than did physicians. The findings indicated also that cardiovascular and respiratory abnormalities were the most common triggers of the system. The presence of the physician was highly significant; however, it did not show any difference in the current study sample. Perhaps, the effect of the physician’s presence in the activation could be detected more in public and university hospitals than in private hospitals.

The findings of the current study should be interpreted taking into considerations the strengths and limitations of the investigation. The study was conducted in four tertiary private hospitals with a relatively good sample size. The limitations include the study evaluation of only adult pre- and post-ICU excluding pediatric ICU and CCU. The study lacks robustness to assumption as the study was conducted in private hospitals in Saudi Arabia where public hospitals may further add to the study findings. Additional studies are needed to confirm the effect of rapid response team system implementation utilizing multisite, cluster-randomized controlled trial designs rather than pre-post implementation designs.

## Conclusion

In conclusion, the implementation of the rapid response team system was significantly associated with a reduction in hospital-wide mortality rate, ICU mortality, and cardiopulmonary arrests. The study demonstrated that the activation of the rapid response team system was associated with the reduction of ICU admission. The findings showed also the practice was effective in improving patient outcomes and quality parameters and facilitating end-of-life care discussions. Further investigations are needed to evaluate the efficacy of the rapid response teams’ system implementation over time and study the specific factors that may diminish its effectiveness.

## Data Availability

The datasets used and/or analyzed during the current study are available from the corresponding author on reasonable request.
